# Improvement of the ayu (*Plecoglossus altivelis*) draft genome using Hi-C sequencing

**DOI:** 10.1186/s13104-023-06362-7

**Published:** 2023-05-30

**Authors:** Masatoshi Nakamoto, Takashi Sakamoto

**Affiliations:** Department of Marine Biosciences, Tokyo University of Marine Science and Technology, Tokyo, 108-8477 Japan

**Keywords:** Ayu, Sweetfish, *Plecoglossus altivelis*, Genome quality, Hi-C

## Abstract

**Objective:**

The ayu or sweetfish *Plecoglossus altivelis* is ray-finned fish that is widely distributed in East Asia. The genome size of ayu was estimated at approximately 420 Mb. Previously, we reported on ayu draft genome assembly by whole-genome shotgun using Illumina short reads and PacBio long reads; however, the assembly was not to chromosome level. Therefore, to improve the draft genome sequence of ayu to chromosome level, we performed *in situ* Hi-C sequencing as a source of linkage information.

**Results:**

The ayu genome assembly yielded 28 large scaffolds that corresponded to the karyotype of ayu (*n* = 28). The resulting ayu genome assembly has a N50 scaffold length of 17.0 Mb, improved from 4.3 Mb. The high-quality reference genome will be helpful for phylogenetic research on bony fishes and for breeding programs in ayu aquaculture.

## Introduction

The ayu or sweetfish *Plecoglossus altivelis* is a ray-finned fish with a wide distribution in East Asia [1–3]. The ayu is a typical amphidromous fish and an economically important aquaculture species in Japan. The species belongs to the teleost group Stomiati and the order Osmeriformes; Stomiati is phylogenetically classified as a sister group of the Neoteleostei. The divergence of Protacanthopterygii (which includes salmon and pike) and the common ancestor of Stomiati and Neoteleostei is estimated to have occurred approximately 190 million years ago [4]. Thus, the ayu holds an important position in teleost fish evolution.

Previously, we reported the ayu draft genome by whole-genome shotgun assembly using Illumina short reads and PacBio long reads [5]. The ayu genome assembly yielded 4,035 scaffolds longer than 1,000 bp. The longest scaffold was 16.8 Mb, with an N50 scaffold length of 4.3 Mb. Scaffolds of the ayu genome assembly were anchored to genetic linkage maps using ALLMAPS [6]; 90.7% of the scaffolds were anchored to linkage maps, and 72.4% were oriented. Thus, the draft genome of ayu was incomplete and the continuity of the assemblies relied on the sequencing and assembly methods.

Hi-C analysis captures the spatial conformation of chromatin [7, 8]. To characterize the three-dimensional architecture of whole genomes, Hi-C detects the physical contacts between chromatin regions through digestion of cross-linked DNA molecules with restriction enzymes and proximity ligation between closely contacted genomic regions. To improve the draft genome sequence of ayu, we performed *in situ* Hi-C sequencing as a source of linkage information and constructed chromosome-level scaffolds.

## Main text

### Materials and methods

The Hi-C sequencing library was constructed using the Proximo Hi-C Kit for Animal Samples (v3.0; Phase Genomics, Seattle, WA) according to the manufacturer’s instructions. As the input sample, 0.3 g of fin tissue obtained from one male ayu individual was rapidly chilled in liquid nitrogen and then ground to a powder. The libraries were quantified using a Qubit dsDNA HS Assay Kit (Thermo Fisher Scientific, Waltham, MA), and the size profile was analyzed on the TapeStation system with the D1000 ScreenTape assay (Agilent, Santa Clara, CA). A library fragment size was approximately 1000 bp. Sequencing of the Hi-C library was carried out on an Illumina HiSeq 4000 system, by Eurofins Genomics K.K. (Tokyo, Japan), with 100-bp paired-end sequencing. The Hi-C data have been deposited in the DDBJ Sequence Read Archive (DRA) under accession number DRA013867. Low-quality bases were removed using the tool Trimmomatic (ILLUMINACLIP:TruSeq3-PE-2.fa:2:30:10 LEADING:15 TRAILING:15 SLIDINGWINDOW:4:15 MINLEN:50) [9]. The Hi-C reads were aligned to the ayu draft genome (DDBJ accession numbers BNHK01000001–BNHK01004035) using Juicer [10]. 36.64% read pairs were mapped that span at least 10 kbp. Candidate chromosomal scaffolds were constructed with the 3D *de novo* assembly (3D-DNA) pipeline with the parameter -r 5 -i 10,000 [11]; the candidate scaffolds were then manually reviewed using Juicebox Assembly Tools [12]. The Hi-C assembly, the linkage map anchored assembly, medaka (*Oryzias latipes*) genome and northern pike (*Esox lucius*) genome were compared using Synima and visualized using Circos [13, 14]. The genome sequences of medaka were obtained from the Ensembl [15].

The genome sequences of northern pike were obtained from the NCBI (accession numbers GCF_004634155).

### Results and discussion

**Fig. 1:**
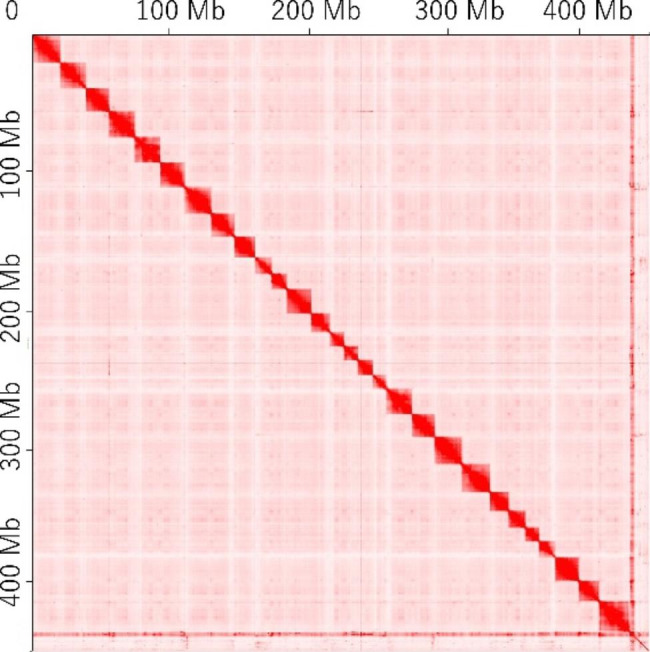
Contact map for the ayu Hi-C scaffolds; the ayu genome assembly scaffolded 28 chromosomes based on Hi-C data. The interaction probability between pairs of genomic regions is indicated using the Juicebox Assembly Tools standard color scheme.

**Fig. 2:**
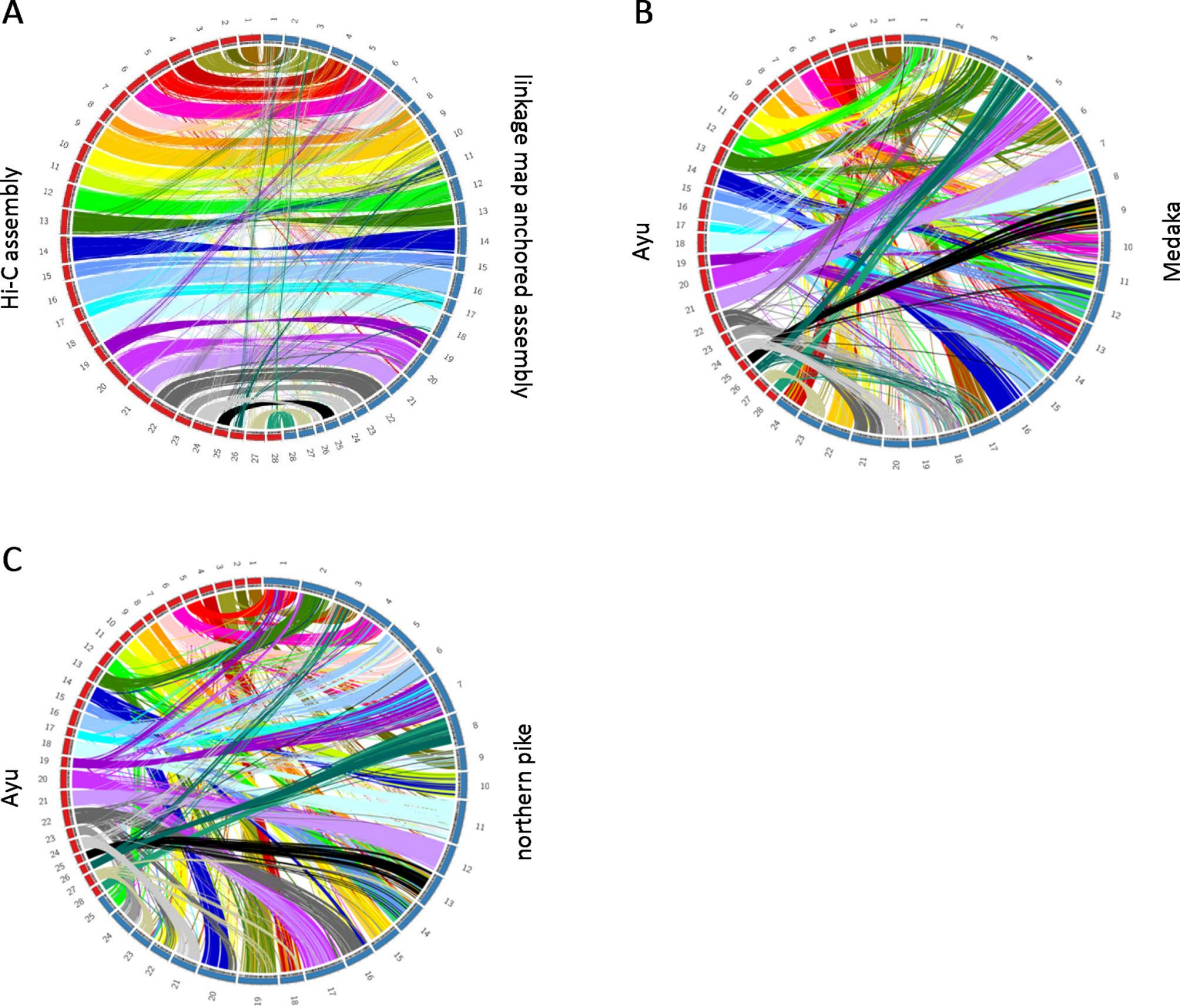
Synteny analysis for ayu Hi-C chromosome. (A) Comparison of the Hi-C assembly with the linkage map anchored assembly of the ayu *Plecoglossus altivelis* genome, visualized with Circos software. Circos plots between Hi-C assembly of ayu and genome of medaka (B) and northern pike (C).

After Hi-C sequencing and quality trimming, 296.1 million paired-end reads (read length 100 bp) were obtained. As a result of scaffolding by Hi-C, the N50 of the ayu genome assembly was improved from 4.3 Mb to 17.0 Mb, and the longest scaffold was improved from 16.8 Mb to 22.4 Mb. Contact maps for the ayu Hi-C data indicated that the ayu genome assembly was constructed of 28 large scaffolds that contained 97.5% (435.7 Mb) of the nucleotides of the draft genome (Fig. 1). The number of large scaffolds corresponded to the karyotype of ayu (*n* = 28) [16]. The improved genome assembly has been deposited in the DDBJ database under the accession number BROE01000001-BROE01004119. Finally, we compared the Hi-C assembly and the previous linkage map anchored assembly and the genome of other teleost fishes. (Fig. 2). These assemblies corresponded almost one-to-one. The major difference was that linkage group 24 and 26 corresponded to Hi-C chromosome 24. Hi-C chromosome 26 was not mapped to the linkage map. Hi-C chromosome 24 corresponded to medaka chromosome 20 and northern pike chromosome 21. Hi-C chromosome 26 corresponded to medaka chromosome 4 and northern pike chromosome 8. Ayu sex determining gene *Amhr2bY* was located in Hi-C chromosome 26, indicating that Hi-C chromosome 26 is sex chromosome of ayu. Further study will be needed to resolve the differences between the Hi-C data and the linkage maps.

Our results show that the ayu genome sequence was scaffolded to chromosome level with Hi-C data. A high-quality reference genome is useful to detect structural variants. We anticipate that our ayu genome assembly will contribute to research on teleost fish evolution as well as to the aquaculture of ayu as a basic food resource.

## Data Availability

The improved genome assembly has been deposited in the DDBJ database under the accession number BROE01000001-BROE01004119.

## Abbreviations

*Mb*: Mega base pair
*DRA*: DDBJ Sequence Read Archive
